# A prospective, multicenter, observational study of complicated skin and soft tissue infections in hospitalized patients: clinical characteristics, medical treatment, and outcomes

**DOI:** 10.1186/1471-2334-12-227

**Published:** 2012-09-25

**Authors:** Benjamin A Lipsky, Gregory J Moran, Lena M Napolitano, Lien Vo, Susan Nicholson, Myoung Kim

**Affiliations:** 1 VA Puget Sound Health Care System & University of Washington, Seattle, WA, USA; 2 Olive View-UCLA Medical Center, Sylmar, CA, USA; 3 University of Michigan, Ann Arbor, MI, USA; 4 Janssen Scientific Affairs, LLC, Raritan, NJ, USA; 5 VA Puget Sound HCS (S- GMS 123), 1660 S. Columbian Way, Seattle, WA, 98108, USA; 6 79 Stone Meadow, Oxford, OX2 6TD, UK

**Keywords:** Complicated skin and soft tissue infections, Prospective observational study, Diabetic foot infection, Abscess, Surgical site infection, Cellulitis, This large prospective observational study that characterized patients with cSSTIs from diverse US inpatient populations provides useful information on the current epidemiology clinical management practices and outcomes of this common infection.

## Abstract

**Background:**

Complicated skin and soft tissue infections (cSSTIs) occur frequently, but limited data do not allow any consensus on an optimal treatment strategy. We designed this prospective, multicenter, observational study to to explore the current epidemiology, treatment, and resulting clinical outcomes of cSSTIs to help develop strategies to potentially improve outcomes.

**Methods:**

From June 2008 to December 2009 we enrolled a pre-specified number of adults treated in 56 U.S. hospitals with intravenous antibiotic(s) for any of the following cSSTIs: diabetic foot infection (DFI); surgical site infection (SSI); deep soft tissue abscess (DSTA); or, cellulitis. Investigators treated all patients per their usual practice during the study and collected data on a standardized form.

**Results:**

We enrolled 1,033 patients (DFI 27%; SSI 32%; DSTA 14%; cellulitis 27%; mean age 54 years; 54% male), of which 74% had healthcare-associated risk factors. At presentation, 89% of patients received initial empiric therapy with intravenous antibiotics; ~20% of these patients had this empiric regimen changed or discontinued based on culture and sensitivity results. Vancomycin was the most frequently used initial intravenous antibiotic, ordered in 61% of cases. During their stay 44% of patients underwent a surgical procedure related to the study infection, usually incision and drainage or debridement. The mean length of stay was 7.1 days, ranging from 5.8 (DSTA) to 8.1 (SSI).

**Conclusion:**

Our findings from this large prospective observational study that characterized patients with cSSTIs from diverse US inpatient populations provide useful information on the current epidemiology, clinical management practices and outcomes of this common infection.

## Background

Skin and soft tissue infections (SSTIs) encompass a variety of conditions, are associated with substantial morbidity, and account for a large percentage of infections requiring hospitalization [1,2]. Infections have generally been considered as complicated (cSSTIs) if they required surgical procedures (in addition to antibiotic therapy) or if they involved deeper subcutaneous tissue (e.g., fascia or muscle) [1,2]. Recently, the U.S. Department of Health and Human Services Food and Drug Administration Center for Drug Evaluation and Research introduced a new terminology for acute bacterial skin and skin structure infections [3], but because this was after we had completed our study we have retained the earlier classification for this report. CSSTIs are now more challenging to manage, as the spectrum of causative pathogens is more complex and the prevalence of antibiotic resistant microorganisms, especially methicillin-resistant *Staphylococcus aureus* (MRSA), has increased [4,5]. While there is currently no clear consensus on how to manage these infections, treatment virtually always includes administering antibiotic therapy and often requires concomitant drainage and debridement procedures. The management approach depends on many factors, including the infection type and severity, epidemiologic setting, likely causative pathogens, and local antibiotic resistance patterns [2,6-8]. Despite the frequent occurrence of cSSTIs, our current understanding is limited about their clinical characteristics at presentation, how they are treated in actual clinical settings, and what the outcomes are of treatment.

Previously published controlled clinical studies of cSSTIs have enrolled relatively homogeneous patient populations, had strict inclusion and exclusion criteria and protocol-defined treatment algorithms [9-13]. They are efficacy trials, designed to determine whether an intervention produces the expected result under ideal circumstances. On the other hand, most published “real-world” studies of cSSTIs are based on retrospective data sources which often do not provide detailed information on patient presentations, approaches to treatment or clinical outcomes [14,15]. Observational studies are designed to examine usual treatment patterns and everyday practices, optimally across a broad spectrum of patients and health care settings, to provide complementary data to those obtained in other study types.

The goal of this prospective observational study of patients with cSSTIs was to explore the current epidemiology, treatment, and resulting clinical outcomes of these infections. A clearer understanding of the current practice patterns in managing patients with cSSTIs in usual clinical settings should help in the development of strategies to improve the treatment of these common and potentially severe infections.

## Methods

We enrolled patients hospitalized between June 2008 and December 2009 in 56 U.S. hospitals that we selected to include those of varying size, ownership status, academic affiliation, and geographic regions. We selected investigators based on their experience working on the inpatient service at each of these hospitals. An Institutional Review Board approved the protocol for each site; 9 sites used a central ethical review board (Quorum Review, Seattle, WA), while the rest used their local Institutional Review Board and all enrolled patients provided informed consent.

### Study population

Patients aged ≥18 years were eligible for this study if they were: hospitalized with signs and symptoms of infection consistent with a diagnosis of diabetic foot infection (DFI), surgical site infection (SSI), deep soft tissue abscess (DSTA), or cellulitis, as defined in the protocol; had an expected inpatient stay for treatment of the study infection of ≥48 h; and, were treated with intravenous antibiotic agents as the primary regimen during the inpatient episode. Patients were excluded from the study if they: had been treated with intravenous antibiotic for >24 h prior to enrollment; were expected to undergo amputation or complete resection of the infected site; had any diagnosis of necrotizing soft tissue infection, burn, gangrene, decubitus ulcer, animal or human bites, known or suspected osteomyelitis, or mediastinitis; were transferred from another hospital within the previous 24 h; were pregnant; had any other known or suspected condition that might jeopardize their adherence to protocol; were employees of, or in another study under, the local investigator or study hospital; and were or wished to be simultaneously participating in any other interventional clinical trial.

Our initial target was to enroll a total of 1,200 patients from all the hospitals combined, with 300 patients in each of the four infection types listed above. To achieve this equal distribution of patients, we closed the enrollment earlier for cellulitis, since we reached the targeted number of patients sooner, and extended the enrollment period for SSI and DFI. After the start of the study, we reduced our target for enrollment for DSTA to 150 patients, in light of the November 2008 FDA Advisory Committee discussion highlighting the limited role of antibiotics in treating these infections [3,10].

### Data collection

We developed a study protocol and standardized case report forms (CRFs) and trained the investigators how to use them. Investigators prospectively collected the required data at the following times: study enrollment; the end of intravenous antibiotic therapy; the end of hospitalization; during a telephone follow-up visit 28–35 days post-discharge from the hospital; and, between these events, as required. They submitted completed CRFs electronically to a centralized database; we then rigorously reviewed them and queried the investigators for any missing or unclear data.

### Study measures

We collected information at the time of enrollment about patient characteristics (e.g., age, gender, ethnicity, race, and source of admission) and co-morbid conditions (e.g., chronic lung disease, diabetes, hepatic dysfunction, peripheral vascular disease, renal insufficiency, and systemic cancer). We defined healthcare-associated infections as those occurring in patients who were: hospitalized during the 6 months prior to the admission; residents of, or admitted from, a nursing home; in an immunosuppressed state; recipients of any antibiotic during the 30 days prior to the study infection; or, receiving dialysis.

We documented the clinical presentation of the infection, including the anatomic site, deepest involvement of the wound, presence of any regional lymphadenopathy, signs and symptoms of inflammation, presence of any abscess, or break in the skin. We estimated wound severity using wound measurements and various infection parameters that comprise an infection scoring system previously developed and validated for DFIs. [16-22] We reported the 5-item wound parameter subtotal, which is identical for open and non-open wounds (range 0–15), as well as the 8-item (range 3–43) and 10-item (range 3–49) wound severity scores for those with an open wound at the end of intravenous treatment. We reported a score as “missing” if wound assessment parameters were incomplete.

We recorded all treatments received for the study infection throughout the hospital stay. This included the initial intravenous antibiotic(s) administered, reasons for their administration and discontinuation, and which if any antibiotics were prescribed at discharge. We recorded all surgical procedures performed related to the study infection, and specifically noted those we considered as “source control” (i.e., incision and drainage, surgical debridement, excision of wound, amputation).

The outcomes we captured included hospital mortality, length of hospital stay (LOS), health-related quality of life scores using EURO-Quality of Life Questionnaire (EQ-5D) index (range 0–1) and EURO-Quality of Life Visual Analog Score (EQ-VAS, range 0–100) [23], the need for any surgical procedures, and clinical assessment of the infection (resolved/cured, improved, unchanged, or worsened, as measured at the end of intravenous antibiotic treatment based on investigator’s clinical judgment). LOS excluded the number of days that discharge was delayed due to a procedure not related to the study infection.

### Data analysis

We conducted descriptive analyses, both overall and by infection type. For patients diagnosed with more than one type of infection, we used the dominant one for classification. We report continuous variables with mean, median and range, and summarize categorical variables using frequencies and percentages.

## Results

### Baseline demographic characteristics

After making adjustments to the target number of patients to ensure that we attained an adequate representation of each infection type, 1,033 patients met the criteria for inclusion in this analysis (Table 1), of whom 278 (26.9%) had DFI, 330 (31.9%) had SSI, 147 (14.2%) had DSTA, and 278 (26.9%) had cellulitis. The overall study population had a mean age of 54.2 years (median: 54.0; range 18–94), 45.9% were female, and 76.6% were white. Patients with DSTA were younger (mean age 44), more likely to be female (57.3%) and to be Hispanic (14.3%), and less likely to be white (64.6%). About three-quarters of the patients were admitted from the emergency department (range 64–83%), and almost one-fifth were admitted from the physician’s office (range 13–23%).

**Table 1:** Demographic characteristics of study population by infection type

	**Overall**	**Diabetic foot infection**	**Surgical site infection**	**Deep soft tissue abscess**	**Cellulitis**
	**(N=1,033)**	**(N=278)**	**(N=330)**	**(N=147)**	**(N=278)**
Age: mean (median, range)	54.2 (54.0, 18–94)	57.6 (57.0, 19–93)	54.9 (55.5, 18–92)	44.0 (43.0, 19–84)	55.5 (53.0, 19–94)
Female: N (%)	474 (45.9)	99 (35.6)	189 (57.3)	53 (36.1)	133 (47.8)
Ethnicity: N (%)					
Hispanic/Latino	80 (7.7)	20 (7.2)	19 (5.8)	21 (14.3)	20 (7.2)
Not Hispanic/Latino	952 (92.2)	257 (92.4)	311 (94.2)	126 (85.7)	258 (92.8)
Missing	1 (0.1)	1 (0.4)	0 (0.0)	0 (0.0)	0 (0.0)
Race: N (%)					
White	790 (76.5)	196 (70.5)	270 (81.8)	95 (64.6)	229 (82.4)
Black	204 (19.7)	64 (23.0)	53 (16.1)	42 (28.6)	45 (16.2)
Asian	13 (1.3)	7 (2.5)	2 (0.6)	3 (2.0)	1 (0.4)
American Indian or Alaska Native	5 (0.5)	0 (0.0)	2 (0.6)	3 (0.7)	2 (0.7)
Native Hawaiian or Pacific Islander	20 (1.9)	10 (3.6)	3 (0.9)	6 (4.1)	1 (0.4)
Missing	1 (0.1)	1 (0.4)	0 (0.0)	0 (0.0)	0 (0.0)
Admission source: N (%)					
Nursing home	4 (0.4)	1 (0.4)	3 (0.9)	0 (0.0)	0 (0.0)
Emergency department	767 (74.2)	225 (80.9)	211 (63.9)	122 (83.0)	209 (75.2)
Physician’s office	188 (18.2)	37 (13.3)	77 (23.3)	20 (13.6)	54 (19.4)
Other	74 (7.2)	15 (5.4)	39 (11.8)	5 (3.4)	15 (5.4)

### Clinical characteristics

**Figure 1:**
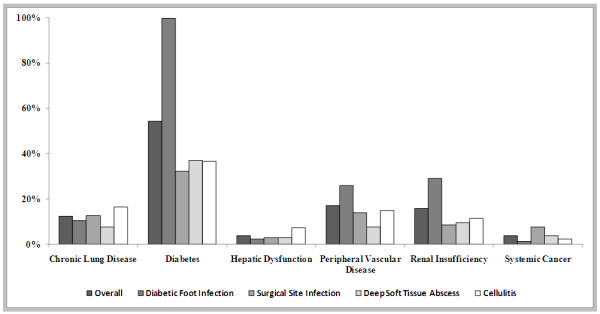
Baseline Co-morbid Conditions of Study Population by Infection Type

**Figure 2:**
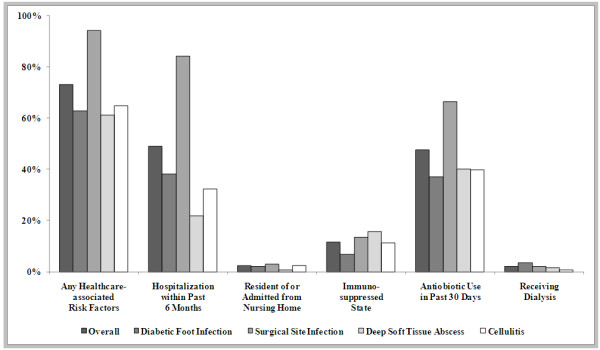
Healthcare associated risk factors in the study population, by infection type

Among the active co-morbid conditions at the time of enrollment (Figure 1), diabetes was the most common in each infection type, followed by peripheral vascular disease and renal insufficiency. As shown in Figure 2, 73.6% of patients had at least one healthcare associated risk factor, the most common of which were hospitalization within the past 6 months and antibiotic use within the past 30 days. Although by definition all SSI cases should be healthcare-associated, we only identified 94.2% as such based on our operational definition, which might miss some cases where the related surgery was performed in an outpatient setting or beyond 6 months.

### Infection clinical presentation

The anatomic site varied by type of infection (Table 2), but occurred most commonly on the foot (28.5%) and lower leg (23.7%). The most common infection site was the foot in DFI patients (87%), the abdomen in SSI patients (29.7%), the lower leg in cellulitis patients (49.6%), and other sites including the hand, arm, face, buttocks in DSTA patients (70.%). In approximately half of the patients, the deepest involvement of the infection site was the subcutaneous tissue. Patients with cellulitis had more infections involving only the epidermis/dermis (43.2%) than those with other infection types. Regional lymphadenopathy was present in 11.0% of patients and was more common among those with DSTA (21.1%). About 90% of patients had erythema, tenderness, pain, or local warmth, the severity of which was mostly moderate (40-50%). Abscess, induration, tenderness, and pain were more common and more severe among patients with DSTA, while patients with cellulitis had more severe erythema and local warmth. Overall, 66.0% of patients had an open wound. We defined “fever” as a temperature of ≥100.4°F (38°C); at baseline fever was present in 85 (8.2%) of the 1033 patients on whom this information was recorded: 8.6% of the patients with cellulitis, 8.6% of those with a DFI, 12.2% of those with a DSTA and 5.8% of those with a SSI. The mean 5-item wound score was 8.4 at baseline and decreased to 3.6 at the end of treatment. The mean 8-item and 10-item wound severity scores were 17.8 and 20.2 at baseline and 12.5 and 13.6 at the end of treatment.

**Table 2:** Baseline clinical presentations of infection site, overall and by infection type

	**Overall**	**Diabetic foot infection**	**Surgical site infection**	**Deep soft tissue abscess**	**Cellulitis**
	**(N=1,033)**	**(N=278)**	**(N=330)**	**(N=147)**	**(N=278)**
Infection site (%)					
Abdomen	11.8	0.0	29.7	6.1	5.4
Upper leg	6.8	0.0	12.1	9.5	5.8
Lower leg^#^	23.7	12.2	16.4	12.9	49.6
Foot	28.5	87.1	6.4	1.4	10.4
Other*	28.5	0.0	33.9	70.1	28.4
Missing	0.8	0.7	1.5	0.0	0.4
Deepest involvement: N (%)					
Epidermis/dermis	274 (23.9)	79 (28.4)	55 (16.7)	20 (13.6)	120 (43.2)
Subcutaneous tissue	575 (55.7)	153 (55.0)	179 (54.2)	101 (68.7)	142 (51.1)
Fascial plane	115 (11.1)	30 (10.8)	56 (17.0)	18 (12.2)	11 (4.0)
Muscle	55 (5.3)	12 (4.3)	33 (10.0)	8 (5.4)	2 (0.7)
Missing	14 (13.6)	4 (14.4)	7 (2.1)	0 (0.0)	3 (1.1)
Regional adenopathy: N (%)					
Present	114 (11.0)	23 (8.3)	20 (6.1)	31 (21.1)	40 (14.4)
Absent	901 (87.2)	252 (90.6)	299 (90.6)	114 (77.6)	236 (84.9)
Missing	18 (1.7)	3 (0.1)	11 (3.3)	2 (1.4)	2 (0.1)
Erythema: N (%)					
Absent	73 (7.1)	20 (7.2)	38 (11.5)	8 (5.4)	7 (2.5)
Mild	218 (21.1)	57 (20.5)	102 (30.9)	26 (17.7)	33 (11.9)
Moderate	516 (50.0)	154 (55.4)	135 (40.9)	76 (51.7)	151 (54.3)
Severe	216 (20.9)	44 (15.8)	49 (14.8)	37 (25.2)	86 (30.9)
Missing	10 (1.0)	3 (1.1)	6 (1.8)	0 (0.0)	1 (0.4)
Abscess: N (%)					
Absent	713 (69.0)	214 (77.0)	244 (73.9)	15 (10.2)^$^	240 (86.3)
Mild	102 (9.9)	30 (10.8)	24 (7.3)	35 (23.8)	13 (4.7)
Moderate	160 (15.5)	25 (9.0)	43 (13.0)	72 (49.0)	20 (7.2)
Severe	46 (4.5)	6 (2.2)	11 (3.3)	25 (17.0)	4 (1.4)
Missing	12 (1.2)	3 (1.1)	8 (2.4)	0 (0.0)	1 (0.4)
Induration: N (%)					
Absent	230 (22.3)	72 (25.9)	89 (27.0)	20 (13.6)	49 (17.6)
Mild	280 (27.1)	75 (27.0)	102 (30.9)	24 (16.3)	79 (28.4)
Moderate	390 (37.8)	105 (37.8)	99 (30.0)	73 (49.7)	113 (40.6)
Severe	120 (11.6)	22 (7.9)	33 (10.0)	30 (20.4)	35 (12.6)
Missing	13 (1.3)	4 (1.4)	7 (2.1)	0 (0.0)	2 (0.7)
Tenderness: N (%)					
Absent	98 (9.5)	55 (19.8)	21 (6.4)	6 (4.1)	16 (5.8)
Mild	265 (25.7)	85 (30.6)	91 (27.6)	21 (14.3)	68 (24.5)
Moderate	445 (43.1)	108 (38.8)	147 (44.5)	63 (24.5)	127 (45.7)
Severe	213 (20.6)	27 (9.7)	64 (19.4)	57 (38.8)	65 (23.4)
Missing	12 (1.2)	3 (1.1)	7 (2.1)	0 (0.0)	2 (0.7)
Pain: N (%)					
Absent	102 (9.9)	56 (20.1)	22 (6.7)	5 (3.4)	19 (6.8)
Mild	228 (22.1)	81 (29.1)	79 (23.9)	14 (9.5)	54 (19.4)
Moderate	425 (41.1)	97 (34.9)	142 (43.0)	62 (42.2)	124 (44.6)
Severe	268 (25.9)	41 (14.7)	82 (24.8)	66 (44.9)	79 (28.4)
Missing	10 (1.0)	3 (1.1)	5 (1.5)	0 (0.0)	2 (0.7)
Local warmth: N (%)					
Absent	94 (9.1)	22 (7.9)	53 (16.1)	9 (6.1)	10 (3.6)
Mild	342 (33.1)	105 (37.8)	136 (41.2)	31 (21.1)	70 (25.2)
Moderate	478 (46.3)	126 (45.3)	109 (33.0)	85 (57.8)	158 (56.8)
Severe	105 (10.2)	21 (7.6)	24 (7.3)	22 (15.0)	38 (13.7)
Missing	14 (1.4)	4 (1.4)	8 (2.4)	0 (0.0)	2 (0.7)
Open wound: N (%)					
Yes	682 (66.0)	255 (91.7)	249 (75.5)	78 (53.1)	100 (36.0)
No	343 (33.2)	21 (7.6)	76 (23.0)	69 (46.9)	177 (63.7)
Missing	8 (0.8)	2 (0.7)	5 (1.5)	0 (0.0)	1 (0.4)
Wound score^: mean (median, range)					
5-item	8.4 (8.0, 0–15)	7.5 (7.5, 0–15)	7.8 (8.0, 0–15)	10.0 (10.0, 0–15)	9.3 (9.0, 2–15)
8-item	17.8 (17, 4–43)	15.7 (15, 4–37)	19.5 (18, 5–43)	20.8 (20, 4–39)	17.0 (16, 8–38)
10-item	20.2 (19, 5–46)	17.8 (18, 5–41)	22.5 (21, 8–46)	23.5 (22.5, 7–43)	18.6 (17, 8–41)

^#^For a diabetic foot infection, infection site would be on the ankle or below.

^*^Other infection sites included face, scalp, neck, chest, pelvis/genitals, back, buttocks, hand, upper arm, and lower arm.

^$^Although DSTA was the dominant diagnosis, the abscess was not present at baseline for some patients (i.e., the abscess developed during hospitalization).

^^^Note: The 5-item wound parameter was estimated for both open and non-open wounds, but was available only for a portion of the total patient population (98.5%). 8-item and 10-item wound severity scores were calculated only among patients with an open wound, but was available only for a portion of patients (83%).

### Treatment patterns

The most commonly used classes of initial intravenous antibiotic prescribed (Table 3) were glycopeptides (i.e., vancomycin) (61.0%), penicillins (37.4%), cephalosporins (18.2%) and lincosamides (14.2%). These antibiotics were administered for 2–3 days on average. Use of glycopeptides was highest in DSTA patients (74.1%), use of penicillins, particularly those combined with beta-lactamase inhibitors, was highest in DFI patients (50.4%), and use of cephalosporins, particularly first-generation agents, was higher in SSI (11.2%) and cellulitis (13.3%) patients than in others. In 88.8% of patients, the investigators administered initial intravenous antibiotic therapy as empiric treatment. In most patients (85.8%), the initial intravenous antibiotic administered was discontinued prior to their discharge. The most common reasons for discontinuation were either switching to another route or formulation (53.6%) or improvement, resolution, or cure of the infection (43.6%). Antibiotic was discontinued for 17.5% of patients because culture results indicated that the initial intravenous antibiotic was not needed, and for 4.1% because the pathogen was not susceptible to the antibiotic agent.

**Table 3:** Antibiotic treatment patterns by infection type

	**Overall**	**Diabetic foot infection**	**Surgical site infection**	**Deep soft tissue abscess**	**Cellulitis**
	**(N=1,033)**	**(N=278)**	**(N=330)**	**(N=147)**	**(N=278)**
Initial intravenous antibiotics: N (%) *					
Glycopeptides^a^	630 (61.0)	167 (60.1)	186 (56.4)	109 (74.1)	169 (60.4)
Penicillins^b^	386 (37.4)	141 (50.7)	101 (30.6)	53 (36.1)	91 (32.7)
Beta-lactamase -inhibitors^c^	380 (36.8)	140 (50.4)	98 (29.7)	53 (36.1)	89 (32.0)
Beta-lactamase -resistant^d^	7 (0.7)	1 (0.4)	3 (0.9)	0 (0.0)	3 (1.1)
Non-beta-lactamase inhibitors^e^	7 (0.7)	2 (0.7)	1 (0.3)	0 (0.0)	4 (1.4)
Cephalosporins^f^	188 (18.2)	41 (14.8)	64 (19.4)	15 (10.2)	68 (24.5)
First-generation	90 (8.7)	13 (4.7)	37 (11.2)	3 (2.0)	37 (13.3)
Third-generation	69 (6.7)	18 (6.5)	15 (4.5)	11 (7.5)	25 (9.0)
Fourth-generation	27 (2.6)	9 (3.2)	13 (3.9)	0 (0.0)	5 (1.8)
Lincosamides^j^	147 (14.2)	31 (11.2)	38 (11.5)	25 (17.0)	53 (19.1)
Fluoroquinolones^k^	90 (8.7)	28 (10.1)	35 (10.6)	11 (7.5)	16 (5.8)
Daptomycin	28 (2.7)	5 (1.8)	13 (3.9)	4 (2.7)	6 (2.2)
Nitroimidazole derivatives^m^	27 (2.6)	7 (2.5)	14 (4.2)	1 (0.7)	5 (1.8)
Other Antibiotics ^n^	26 (2.5)	8 (2.9)	8 (2.4)	4 (2.7)	6 (2.2)
Treatment duration of the most common initial intravenous antibiotics: mean (median, minimum-maximum)					
First-generation cephalosporins	2.5 (1.7, 0–27)	1.5 (1.7, 0–3)	3.2 (1.7, 0–27)	1.6 (1.5, 0–3)	2.2 (1.5, 0–12)
Glycopeptides	3.2 (2.6, 0–24)	3.7 (2.7, 0–24)	3.1 (2.6, 0–16)	2.8 (2.6, 0–11)	3.0 (2.7, 0–14)
Lincosamides	2.2 (1.6, 0–10)	1.9 (1.2, 0–8)	2.5 (1.5, 0–10)	2.3 (2.3, 0–7)	2.1 (1.7, 0–9)
Penicillins (beta-lactamase-inhibitors)	3.4 (2.6, 0–36)	3.8 (2.8, 0–24)	3.9 (2.9, 0–36)	2.4 (2.1, 0–7)	2.8 (2.4, 0–13)
Reason for administration of initial intravenous antibiotics: N (%)*					
Empiric treatment prior to culture test results	917 (88.8)	243 (87.4)	292 (88.5)	137 (93.2)	245 (88.1)
Not responding to previous antibiotic treatment	42 (4.1)	6 (2.2)	13 (3.9)	6 (4.1)	17 (6.1)
Other	64 (6.2)	13 (4.7)	24 (7.3)	6 (4.1)	21 (7.6)
Reason for discontinuation of initial antibiotics: N (%)*	886	232	271	136	247
Switched to another route/formulation^	475 (53.6)	108 (46.6)	147 (54.2)	82 (60.3)	138 (55.9)
Infection improved, resolved, or cured^	386 (43.6)	105 (45.3)	99 (36.5)	62 (45.6)	120 (48.6)
Culture results indicate this antibiotic agent not needed	155 (17.5)	43 (18.5)	56 (20.7)	32 (23.5)	24 (9.7)
Pathogen not susceptible to this antibiotic agent^	36 (4.1)	8 (3.4)	19 (7.0)	3 (2.2)	6 (2.4)

*Not mutually exclusive.

^Percentages reported based on the number of patients who discontinued.

^a^Includes vancomycin; ^b^Includes c, d, e below; ^c^Includes amoxi-clavulanico (amoxicillin, clavulanic acid), clavucar (clavulanic acid, ticarcillin), duocid (ampicillin, sulbactam), piperacillin w/tazobactam; ^d^Includes dicloxacillin, nafcillin, oxacillin, piperacillin; ^e^Includes amoxicillin, ampicillin, benzylpenicillin; ^f^Includes g, h, i below; ^g^Includes cefadroxil, cefalexin, cefazolin; ^h^Includes cefixime, cefotaxime, cefpodoxime, ceftazidime, ceftriaxone; ^i^Includes cefepime; ^j^Includes clindamycin; ^k^Includes ciprofloxacin, levofloxacin, moxifloxacin; ^l^Includes daptomycin, linezolid; ^m^Includes metronidazole; ^n^Includes linezolid, tigecycline, aztreonam, azithromycin, erythromycin, rifampicin, rifaximin, doxycycline, minocycline.

### Procedures and outcomes

A total of 41.2% of patients underwent surgical procedures related to the study infection (Table 4), many of which were performed on those with DSTA (68.0%). Overall, 17.5% of patients had a source control procedure, with incision and drainage being the most common (11.3%).

**Table 4:** Surgical procedures performed and patient outcomes by infection type

	**Overall**	**Diabetic foot infection**	**Surgical site infection**	**Deep soft tissue abscess**	**Cellulitis**
	**(N=1,033)**	**(N=278)**	**(N=330)**	**(N=147)**	**(N=278)**
Surgical procedures related to study infection: N (%)	426 (41.2)	123 (44.2)	148 (44.8)	100 (68.0)	55 (19.8)
For source control: N (%)	181 (17.5)	56 (20.1)	48 (14.5)	56 (38.1)	21 (7.6)
Incision and drainage	117 (11.3)	25 (8.9)	29 (8.8)	47 (32.0)	16 (5.8)
Surgical debridement	41 (3.9)	11 (3.9)	18 (5.5)	8 (5.4)	4 (1.4)
Excision of wound	5 (0.4)	3 (1.1)	0 (0.0)	1 (0.7)	1 (0.4)
Amputation	18 (1.7)	17 (6.1)	1 (0.3)	0 (0.0)	0 (0.0)
Clinical assessment: N (%)					
Resolved/cured	82 (7.9)	22 (7.9)	15 (4.6)	12 (8.2)	33 (11.9)
Improved	836 (80.9)	213 (76.6)	274 (83.0)	131 (89.1)	218 (78.4)
Unchanged	32 (3.1)	10 (3.6)	13 (3.9)	0 (0.0)	9 (3.2)
Worsened	6 (0.6)	3 (1.1)	2 (0.6)	0 (0.0)	1 (0.4)
Missing	77 (7.5)	30 (10.8)	26 (7.9)	4 (2.7)	17 (6.1)
Quality of life^					
Change in EQ-5D Index at discharge: mean (median, range)	0.1 (0.1, -0.8-0.9)	0.1 (0.1, -0.8-0.9)	0.1 (0.1, -0.7-0.9)	0.2 (0.2, -0.3-0.7)	0.2 (0.1, -0.7-0.8)
Change in EQ-VAS at discharge: mean (median, range)	11.7 (10, -93-100)	11.4 (10, -93-92)	11.5 (10, -55-90)	13.7 (10, -45-100)	11.1 (10, -50-85)
Length of stay: mean (median, range)	7.1 (5, 1–55)	7.6 (6, 1–26)	8.1 (6, 1–55)	5.8 (5, 2–31)	6.0 (5, 1–35)
Hospital mortality: N (%)	4 (0.4)	2 (0.7)	2 (0.6)	0 (0.0)	0 (0.0)

^Note: Quality of life scores were calculated only for a portion of the total patient population with available data (99.4% for EQ-5D Index at baseline, 89.7% for change in EQ-5D Index at discharge, 99.3% for EQ-5D VAS at baseline, and 89.5% for change in EQ-5D VAS at discharge).

At the end of intravenous treatment, the infection was classified as resolved/cured in only 8% of patients but improved in 80.9%. Health-related quality of life at baseline was similar across infection types, with a mean EQ-5D index of 0.6 and mean EQ-VAS of 57.4. On average, EQ-5D index increased 0.1 from baseline to hospital discharge, with a greater improvement among DSTA and cellulitis patients. Mean change in EQ-VAS was 11.7, with a greater improvement among DSTA patients. The mean LOS was 7.1 days, ranging from 5.8 days for DSTA patients to 8.1 days for SSI patients. Hospital mortality was only 0.4%.

## Discussion

We initiated this multicenter, prospective observational study to learn about the current epidemiology, clinical presentation, treatment and outcomes for patients with cSSTIs. The study protocol did not recommend or mandate any initial or subsequent interventions, but CRFs recorded each of the local physicians’ management decisions. The prospective design of the study made it possible to capture a more comprehensive patient profile, including data on risk factors for healthcare-associated infections, wound measurements, and infection parameters. These enabled us to score wound severity, to clinically assess the infection, to note the reasons investigators initiated and discontinued each agent, and to evaluate quality of life measures. Furthermore, we enrolled a large number of patients with varied types of infections admitted to hospitals representing a wide-range of types of institutions and geographic regions. Thus, these data are likely to reflect usual current clinical practice for managing cSSTIs in hospitalized patients in the US.

Our study showed that, among the 1,033 patients enrolled, 73.5% had a healthcare-associated infection. This is nearly identical to the 73.6% reported in a single-center retrospective cohort study of patients hospitalized with cSSTIs [15]. Compared to our study, their criteria for defining healthcare-associated infection used a longer time frame for previous hospitalization (1 year versus 6 months) and previous antibiotic use (90 days versus 30 days) and did not include immunosuppressed state as a criterion. This rate of healthcare associated infection is considerably higher, however, than the 27% reported in a recent retrospective cohort study of more than 12,000 patients hospitalized with skin, soft-tissue, bone or joint infection [24]. This difference could be attributed to that study including bone and joint infections, and using a definition of healthcare associated infection with a substantially shorter timeframe for previous hospitalization compared with ours (30 days versus 180 days) and that did not include previous antibiotic treatment, a criterion met by 47.6% of our patients. Physicians treating patients with cSSTI may find the high prevalence rate of healthcare-associated infections noted in this study to be clinically important, as previous studies [15,24] have reported that these patients had longer lengths of stay, higher mortality, and higher hospital costs.

Our evaluation of initial antibiotic treatment revealed that vancomycin was used considerably more often (in 61.0%) than has been reported in previous studies. Two retrospective multicenter studies performed in the past decade, both of which used an inpatient claims database, eported initial treatment with vancomycin in 17.6% and 27.8% of cSSTI patients, respectively [14,25]. The much higher use of vancomycin in our study is likely attributable to the steep rise in the prevalence of MRSA as a pathogen in cSSTIs over the past several years [26]. It is noteworthy that the initial intravenous therapy selected was discontinued in over a fifth of patients because it was either not needed or inappropriate. Also of note is that a substantial percentage of patients were treated with relatively broad-spectrum agents that are typically used to cover gram-negative bacilli (e.g., fluoroquinolones, 3^rd^ generation cephalosporins, aminoglycosides), which is probably unnecessary in most cases of cSSTI.

The mean LOS we observed in this study is longer than might be observed in some practices; this is probably related to the fact that we enrolled patients with more severe infections as they needed to be hospitalized for at least 48 h. Our LOS is, however, similar to those previously reported in patients hospitalized for cSSTIs [15,24,27]. Despite differences in patient population, the overall mean LOS in our study (7.1 days, ranging from 5.8 to 8.1 days) was similar to that reported by Lipsky et al. (7.3 days, ranging from 5.1 to 8.9 days depending on infection type and whether or not the infection was complicated) [24]. The LOS for patients with cellulitis or SSI, the two infection types examined in our study that were also included individually in the Lipsky study [24], were slightly higher in our study, probably because many of our patients would have been considered to have a complicated infection. The LOS found in our study is also similar to that found by Zilberberg et al. [15], (9.4 days for patients with healthcare associated infection and 5.5 days for community associated infection) and Edelsberg et al. [14] (5.2 days for those successfully treated and 10.6 days for those who failed treatment). The in-hospital mortality rate in the overall cohort in this study (0.4%) is, however, considerably lower than the rates reported in the literature (0.8%-5.2%) [14,15,24,27,28]. This could be related to differences in patient populations, infection types or severity, or how the infections were managed.

Our study has several limitations. First, there is a possibility of selection bias in the patients enrolled or hospitals selected, notwithstanding our considerable efforts to minimize this by establishing *a priori* definitions and standardized data collection procedures. Secondly, we did not recognize the need to provide the investigators with specific enough criteria to identify DFI or SSI until after patient enrollment had begun. Initially, we intended for the investigators to use their discretion when enrolling patients. While using this method would have added to the naturalistic design of the study and resulted in enrollment of a more heterogeneous population, several sites asked us questions about what constituted a DFI or SSI. Thus, we sent out clarification to all the sites on how to define these infection types (see the appendix). Third, although the data from this study underwent a rigorous review and query process, the data accuracy and completeness ultimately depended on the investigators at the study sites. To minimize this problem, we made efforts to sign up experienced clinicians and trained them on data collection in advance of the study. Fourth, clinical outcome was based on the investigators’ subjective assessment and not on a change in the wound score or microbiological response at the end of therapy. This was necessary, as there are no agreed upon criteria for the wound score and the presence and resolution of cSSTIs are based on clinical (not microbiological) criteria. Finally, our findings may not be generalizable to non-hospitalized patients, to patients hospitalized in other countries, to other types of cSSTIs not included in our study, or to patients who had conditions that were excluded from our study.

Strengths of this study include the prospective observational design, the variety of hospital types, the training of the investigating clinicians, the attempt to define types of cSSTIs, the development of standardized CRFs, the use of a validated wound scoring system and the large number of patients enrolled.

## Conclusions

This multicenter, prospective observational study of patients with cSSTIs was designed to explore the current epidemiology, treatment, and resulting clinical outcomes of patients hospitalized with these infections. During an 18 month period we enrolled 1033 patients. Almost 3/4ths of patients had at least one healthcare associated risk factor. Infections of the lower extremity comprised over half the cases and two-thirds has an open wound. In almost 90% of cases antibiotic therapy was initially an empiric intravenous agent, the most common of which was vancomycin. A substantial percentage of the patients were treated with unnecessarily broad-spectrum agents. Empiric antibiotics were discontinued in about 1/5th of patients because culture results indicated they were not needed or the pathogen was not susceptible to the selected agent. Over 40% of patients underwent some form of surgical treatment. At the end of therapy infection was improved or resolved in almost 90% of patients. Because of the study design these data likely reflect usual current clinical practice for managing cSSTIs in hospitalized patients in the US. This study provides useful information on the current epidemiology and clinical management practices and outcomes of hospitalized patients with cSSTIs.

## Appendix

### Appendix: clarification of DFI and SSI definitions*

In order for a patient to be enrolled as a diabetic foot infection (DFI) patient, they were required to meet all of the following 3 conditions:

· Have a history of diabetes mellitus

· Have an infection on or below the ankle

· Have a break (i.e., ulcer) in the epidermis where the infection started (“You must be able to answer “yes” to “open wound” on the Baseline Characteristics of Open Wound assessment)

· Have a purulent discharge or at least two of the following (on the Baseline Signs and Symptoms of Inflammation assessment):

○ Erythema

○ Pain

○ Tenderness

○ Local warmth

○ Induration

In order for a patient to be enrolled as a surgical site infection (SSI) patient, they were required to meet all of the following 3 conditions:

· Have an infection that occurred within 30 days after an operation

· Have an infection that involves only skin or subcutaneous tissue at the incision site

· Have at least one of the following:

· Purulent drainage, with or without laboratory confirmation, from the surgical incision

· Organisms isolated from a culture of an aseptically obtained specimen of wound fluid or tissue from the surgical incision site

· At least two of the following signs or symptoms of infection: pain or tenderness, localized swelling, redness, or warmth

· Diagnosis of surgical incisional infection by the operating surgeon or attending physician

· An abscess or other evidence of infection involving the surgical site found on direct examination, during reoperation, or by histopathologic or imaging examination

The following conditions were not to be reported as SSI:

· Stitch abscess (minimal inflammation and discharge confined to the points of suture penetration)

· Infection of an episiotomy or newborn circumcision site

· Infected burn wound or infected stab or traumatic wound

*These specific guidelines for DFI and SSI patients were issued June 25, 2009. Following database lock, it was discovered that of the 278 enrolled DFI patients, 23 were not indicated as having an open wound. 19 of these were enrolled prior to the June 25, 2009 guideline memo. None of these 23 DFI patients were excluded from analyses on the grounds of their not having an open wound.

## Competing interests

BAL, GM, and LN have served as consultants to Janssen Scientific Affairs, LLC, LV, MK, and SN are employees and shareholders of Janssen Scientific Affairs, LLC.

## Authors’ contributions

BAL was involved in designing the study, developing the protocol and case report forms, reviewing and interpreting the data, writing and revising the manuscript. GJM was involved in designing the study, developing the protocol and case report forms, reviewing and interpreting the data, and helping to write and review the manuscript. LMN was involved in designing the study, developing the protocol and case report forms, reviewing and interpreting the data, and helping to write and review the manuscript. LV helped secure financing for the study and oversaw recruitment of sites, as well as being involved in designing the study, developing the protocol and case report forms, reviewing and interpreting the data, and helping to write and review the manuscript. SCN involved in the design of the study, development of the protocol and case report form and interpretation of the data. MK helped secure financing for the study and oversaw recruitment of sites, as well as being involved in designing the study, developing the protocol and case report forms, reviewing and interpreting the data, and helping to write and review the manuscript. All authors read and approved the final manuscript.

## Funding

The work was supported by Janssen Scientific Affairs, LLC, Raritan, NJ, USA.

## Pre-publication history

The pre-publication history for this paper can be accessed here:

http://www.biomedcentral.com/1471-2334/12/227/prepub
